# Sir Sydney Waterlow's Paper

**Published:** 1888-12-08

**Authors:** B. Burford Rawlings


### Sir Sydney Waterlow's Paper.

### AN EXAMINATION OF SOME OF ITS CONTENTS.

We must not make it a complaint that Sir Sydney Waterlow's paper contains little that is novel. The product of one who commands exceptional advantages and is naturally accepted as an authority, it adds hardly anything to our knowledge, for the simple reason that the hospital theme is wellnigh exhausted. Nevertheless, much in this paper is welcome because it is true, and even truth is not independent of iteration. Sometimes error is perpetuated by similar means, and it is because a few venerable errors appear to have crept into Sir Sydney's paper, and have received the sanction of his endorsement, that we venture to question statements which otherwise might have been treated as already disposed of.

With a plain eulogy of the Hospital Sunday Fund and the proposition that "its influence on the medical charities of the Metropolis" had been beneficial, there would have been general agreement. We are only too glad to witness a censorship of some sort, exercising control, however indirectly, over the heterogeneous and motley rout of Metropolitan medical charities, to inquire too clearly into its charter, or to affect too much concern for its occasional errors. All must hope the Hospital Sunday Fund will go on and prosper.

But in eulogising the Sunday Fund, was it necessary to make the merits of its management manifest by contrasting them with hospital defects? Comparisons are odious because they are seldom reasonable. What is the value of comparison when the conditions are essentially different? We should not rate very highly the contention that red and white are not alike, but should we think of the deduction that because red is not white therefore it is black?

Yet this is the method of reasoning which appears to be employed in order to show that contributions ought to be sent to the Hospital Sunday Fund rather than to individual hospitals. The writer says;

"Many will perhaps be suprised to learn that from the returns annually sent to the Mansion House it is evident that, as a rule, of every 20*s*. given at bazaars or public dinners in aid of medical charities, a sum of from 4*s*. to 6*s*. has to be deducted for expenses of collection, and never reaches the suffering objects of those charities. In the case of charities frequently advertised in our daily and weekly journals, the percentage of deduction is very little less. Of the money received on Hospital Sunday less than 10*d*. in 20*s*. is spent in collection and distribution, including all salaries, advertising, etc."

Sir Sydney Waterlow is the respected treasurer of the only fully endowed hospital in the metropolis. His mind has never been exercised about ways and means in respect of his own grand charity, and it seems scarcely possible, therefore, that he can altogether realise the difficulties and the duties of managers to whom every day is a day of financial care and perplexity. Is it quite fair that one so placed should sit in judgment upon the financial management of institutions kindred in work with his own, but alien to the chief condition which makes the carrying on of that work a task of unqualified satisfaction? How can one thus situated understand the radical differences between the labour of raising money by such machinery as the Hospital Sunday Fund has at command, and by the efforts of individual hospitals?

With regard to the figures given, we think there must be some misunderstanding. Bazaars are, perhaps, more costly than any other method of raising money, and it is a pity they are so common. But this much may be said: they often bring in help which would not otherwise be obtained. Putting bazaars on one side, it is not usual, we think, for well-managed hospitals to expend from 4*s*. to 6*s*. in the pound upon collecting funds. Perhaps Sir Sydney takes the whole expenses of "management," as grouped in the Sunday Fund returns, to represent expenditure upon appeals—a curious but not an unheard of mistake. Whatever the explanation, we are concerned with the principle of the comparison rather than with the actual figures.

In the first place, the Sunday Fund starts with the distinct advantage which pertains to its representative character, while the sum chargeable to expenses does not include the payments by the clergy, many of whom make a special effort to arouse their congregations. In the case of individual hospitals appealing for funds, every expense must be met. Tavern keepers cannot be expected to provide the dinner except as an affair of profit, neither will a glee party or a military band attend without payment. When appeals are issued it must be through the post, and, in short, hospitals must conduct their arrangements in a purely business way. To place the Sunday Fund upon the same footing, it would be necessary that its council should hold a position no more commanding than that of a hospital committee. Rent would have to be paid for every church, and fees to the clergy, the organist, and the choir. The large outlay incurred, for instance, by the *Lancet*, whose special issues have been, we believe, very costly affairs, would have to be included, together with the expense of other similar advocacy which is forthcoming in a variety of quarters for a central organisation, but would certainly not be afforded to any single charity.

No doubt much of the force of these observations would be lost if the Sunday Fund were capable of providing the whole annual sum the hospitals require. At present the grants to the several hospitals represent from 4 to 8 per cent. of the expenditure. An interesting and strictly pertinent sum might be formulated thus: if it costs 10*d*. in the pound to raise £40,000, what would it cost to raise £160,000? although we do not think this calculation is capable of being worked out by any rules of arithmetic.

Moreover, the Hospital Sunday managers are in the happy position of contributors. They give whatever is placed in their hands for the purpose, and need to give no more. Hospital authorities are less fortunate. Their institutions require each a certain sum, and unless they are prepared to diminish their work they know it must be obtained. The methods they employ, and the expenses they incur, are not voluntary, but enforced. Where is the hospital manager so infatuated with a love of warring against difficulty and famine that he would not welcome as a saviour the organisation which should give his institution peace and plenty? If such an organisation ever did exist, there would be no longer any place for those diatribes concerning the zeal of successful managers and secretaries, without which no essay about hospitals is complete. We know what it is "to damn with faint praise," but Sir Sydney will have no praise at all, and opines that "pecuniary success" . . . . is perhaps more the fault of the contributors . . . . than of the secretaries."

Yet we entirely uphold the principle enunciated, and disagree with the writer only because he fails to be articulate and discriminating. There is perfect truth in the lament that cleverness in the manner of appealing tells more than intrinsic merit in the institution. But we may fairly demand of the critic that he shall be explicit. Sir Sydney is of course alluding to special hospitals. Which of them, then, is he prepared to uphold, and which to condemn? He says he does not object to "hospitals for consumption, for contagious diseases, or for lying-in;" but do these exhaust the list of exceptions, and is it meant that there may be any number of hospitals devoted to the wants of these three classes of patients? We venture to think that the chief evil is not so much in the many varieties of special hospitals as in the multiplication of institutions for treating the same maladies, and that it is less the general hospitals which are aggrieved than the high-class special hospitals whose titles are imitated and their objects parodied by worthless institutions. Were some enactment possible to compel every "hospital" appealing for funds to state plainly the number of beds it has in occupation, a check would be placed upon the evil, and much confusion would be prevented.

If the general hospitals have tardily provided many special departments for the treatment of particular diseases, is it not because the special hospitals have reminded them of their duty? And let the former be as comprehensive as they may, groups of maladies will still remain which competent authorities tell us can be better studied and better treated in separate institutions. Again, this indiscriminating argument for the abolition of special hospitals seems always conceived in the professional interest as opposed to that of the patients! Suppose it be true that for clinical purposes a sufficient assortment of every variety of disease is to be found in the wards of a general hospital, are we to forget the first duty of a hospital—the relief of present suffering? What is to become of the great body of patients who are the victims of maladies only sparingly admitted to general hospitals? Having furnished a few specimens for demonstration are the rest to be refused on the ground that they are superfluous.

This is not an imaginary picture. It is what has been, is, and must be. The history of metropolitan medical charity is a volume of splendid record, but to those who can read between the lines there runs through it a tale of terrible omission and neglect. What was it gave rise to the establishment of the chief special hospitals? Undoubtedly a conviction that whole classes of sufferers were virtually excluded from treatment, and left to die like dogs; and it is not because certain persons have promoted a number of petty and valueless institutions that the *raison d'être* of the great special hospitals is to be called in question. That would be but poor logic, and poorer humanity. The origin of the Consumption Hospital at Brompton, and of the National Hospital for the Paralysed and Epileptic, Queen Square—taking two of the chief examples of specialism—had nothing questionable about it. These institutions were established, each in its turn, to meet a crying want which it is no reflection upon the general hospitals to say they were unable to supply, and they have gone on and prospered because the value of their work is known and appreciated.

But we do not stop here. We give philanthropy the first place, but we join issue along the whole line. The work of special hospitals of the right sort could not fail to have a high scientific value, and if hitherto the opportunities they have provided for the study of particular groups of maladies have not been taken advantage of, it is not because they have been unwilling to open their doors to students, but because of the jealousy with which they have been regarded by the general hospitals and the professional pressure which has been brought to bear upon their staffs. Special hospitals would have been willing, without doubt, to affiliate themselves for teaching purposes to the established medical schools, as we are now told should be done, but suggestions to this end have been treated with disdain. The representative authorities of general hospitals have never been willing to acknowledge that the special hospitals were able to supply anything not already possessed, and, unfortunately for science no less than humanity, the physicians and surgeons attached to the special hospitals, and who mostly hold general hospital appointments too, have found difficulties not of their own making about utilising to the full and on the spot the valuable opportunities surrounding them.

Yet it would be easy to show by irreproachable evidence what copious contributories some of the special hospitals have been to the riches of medical science. The knowledge gleaned in their wards has been conveyed, almost *sub rosâ*, to the students of the general hospitals, or has been communicated to the medical world in many a text-book. In the current number of the *University Medical Magazine* (Philadelphia), appears a paper written by an American physician to show the pre-eminence of London over all other cities as a place for clinical study, and it is noteworthy that of the five institutions which he puts forward in illustration of the teaching power of our metropolis, three are special hospitals—the two we have already mentioned, and the Hospital for Sick Children.

Besides the living testimony afforded by a constant attendance in large numbers of qualified, and often distinguished, practitioners in the wards, and at the out-patients' practice of some of the special hospitals, the proposals of the Post Graduate Association afford ample proof that the value of these institutions for teaching purposes is becoming more fully recognised, and if the important scheme now on foot be carried through, the material they yield will be at last systematically utilised. Performing splendid work already, notwithstanding the disabilities sought to be put upon them, they will then rise to the level of their opportunities; and although they must always surrender premier place to their greater brethren, they may fairly look to be regarded as worthy co-labourers in a field where there is ample work for all.

B. Burford Rawlings.

